# Efficacy of Blackleg Major Resistance Genes in *B. napus* in Germany

**DOI:** 10.3390/pathogens11040461

**Published:** 2022-04-12

**Authors:** Dima Alnajar, Andreas von Tiedemann, Birger Koopmann

**Affiliations:** Plant Pathology and Crop Protection, University of Goettingen, 37077 Goettingen, Germany; atiedem@gwdg.de (A.v.T.); bkoopma@gwdg.de (B.K.)

**Keywords:** *L. maculans*, *L. biglobosa*, *B. napus*, oilseed rape, blackleg disease, phoma stem canker, qualitative resistance, major resistance genes

## Abstract

*Leptosphaeria maculans* is one of the major pathogens of oilseed rape (*B. napus*). It causes blackleg disease, which accounts for significant yield losses worldwide. Using cultivars that harbor major resistance (*R*) genes is one of the most effective control methods. However, the efficacy of major *R* genes is related to the frequency of the corresponding avirulence (*Avr*) genes in a *L. maculans* population. In this paper, we report the *Avr* profiles of *L. maculans* populations and the ratio of its mating types in Northern and Central regions of Germany. Eleven *Avr* genes in five-hundred and seventy-four isolates were characterized either by applying cotyledon tests on a *B. napus* differential set or by amplifying avirulence gene-specific PCR markers. Fifty-two races were determined, among which the most dominant race was *Avrlm6, -7, -11, AvrlepR1, -R2*. Results showed that the resistance gene *Rlm2* is 100% ineffective, some other major *R* genes such as *Rlm1, Rlm3*, *Rlm4* and *LepR3* are partially effective (with corresponding *Avr* frequencies ≤ 42%), while *LepR1*, *LepR2*, *Rlm6*, *Rlm11* and *Rlm7* can still provide relatively effective resistance in the German fields investigated (with corresponding *Avr* frequencies of 63–100%). Sexual reproduction is a factor that enhances the potential of *L. maculans* to evolve under selection pressure. Mating types of the *L. maculans* populations did not deviate from the ratio of 1:1 in the examined regions, indicating that sexual reproduction and ascospores play central roles in the *L. maculans* lifecycle. Overall, this study provides an important dataset for the establishment of a strategic plan to preserve the efficacies of major *R* genes in Germany by applying cultivar rotations of oilseed rape.

## 1. Introduction

Blackleg disease (phoma stem canker) is an economically important disease in many oilseed rape-growing areas in the world [1]. The causal agent of the disease is a two-species complex: *Leptosphaeria maculans* (anamorph = *Plenodomus lingam*) and *Leptosphaeria biglobosa* (anamorph = *Plenodomus biglobosus*) [2,3]. Both species coexist in the field. However, the ratio of *L. maculans* to *L. biglobosa* in a region is decisive for disease severity, so that *L. maculans* is mainly responsible for significant yield losses [4]. *L. maculans* was reported to be dominant in Germany and other western European countries [1].

Having a complex lifecycle with two reproduction systems and different dispersal mechanisms, *L. maculans* has a high evolutionary potential that allows it to readily adapt to new conditions, such as introducing new *R* genes in its host plant [5]. Generally, the fungus survives saprophytically on stubbles of a previous season by means of the sexual fruiting bodies, pseudothecia. Once mature, pseudothecia eject wind-borne ascospores, resulting in a primary infection of host plants. Ascospores germinate on cotyledons and young leaves. Hyphae grow biotrophically to form characteristic phoma lesions, mostly with asexual fruiting bodies, pycnidia. The latter contain pycnidiospores, which can be spread by rain splashes. Their spread may finally result in a secondary infection. The fungus grows symptomless from leaves through petioles to stems. Once in the stem base, the fungus turns necrotrophic and causes canker [1,6]. Additionally, *L. maculans* can be seed-transmitted [7]. Studies describing a high diversity in *L. maculans* populations have repeatedly emphasized the importance of sexual reproduction for generating wide genetic variation [8,9]. However, despite its importance, sexual reproduction might not be dominant in some seasons. Such cases were reported in western Canada, where ascospores were not detected in the air in some years, although infection was severe. Thus, it was concluded that ascospores did not form the main inoculum. Instead, pycnidiospores represented the alternative main source of inoculum [10]. *L. maculans* has two mating types, *MAT1-1* and *MAT1-2* [11]. According to the random mating hypothesis, the mating type frequencies in a population should be 1:1 under random sexual outcrossing [12]. Defining the idiomorph ratios in a *L. maculans* population helps to determine the source of the primary inoculum, and thus the speed expected for the pathogen to evolve new races in a specific region. 

Integrated management of phoma stem canker includes stubble management, crop rotation, applying fungicides and using resistant cultivars [13,14]. Two types of resistance are known: polygenic, non-race specific, quantitative resistance and major gene, race-specific, qualitative resistance [1,15]. The immune system in plants is described by a “Zigzag” model of several phases [16]. Once *L. maculans* interacts with the host, the fungus initially confronts extracellular pattern recognition receptors (PRRs) which recognize pathogen-associated molecular patterns (PAMPs), and this recognition results in PAMP-triggered immunity (PTI). The pathogen excretes specific effectors to suppress PTI. If the plant cannot recognize these effectors, an effector-triggered susceptibility (ETS) will be initiated. Otherwise, a specific recognition of effectors will activate an effector-triggered immunity (ETI) [16]. Effector-specific recognition happens according to the gene-for-gene concept, in which each *Avr* gene in the pathogen has a counterpart major *R* gene in the host [17,18]. The classic perception of the recognition process depicts it as a direct interaction between a plant receptor protein and a pathogen avirulence protein [18,19]. However, a more contemporary point of view speculates that *R* gene(s) in a plant monitor the occurrence of modulations of host cell components that are targeted by the pathogen to prepare the cell environment for the invasion [20,21]. Through the co-evolution of pathogens and their hosts, natural selection allows a pathogen population to modify their *Avr* profile to generate effectors able to successfully suppress PTI [16].

New emerged isolates have previously been categorized into pathogenicity groups, including *L. biglobosa,* until Shoemaker and Brun [2] provided the final taxonomic evidence to separate *L. biglobosa* as a distinct species. Since then, there has been a considerable gain of knowledge about major blackleg resistance genes, which have also been incorporated into actual tester sets, allowing the definition of races (up to 2^n^). Characterization of *L. maculans* by their races was suggested by Balesdent et al. [22]. This characterization relies on differential cotyledon reactions of tester lines to individual isolates of *L. maculans* based on *Avr*–*R* gene interactions. *Avr*–*R* gene interactions in the *L. maculans*-*B. napus* pathosystem can be more complex than involving two genes. On the one hand, a redundant recognition of a single *Avr* gene can be displayed by two major *R* genes [23]. Larkan et al. demonstrated that the avirulence gene *Avrlm1* encodes effectors that can trigger the two major *R* genes *LepR3* and *Rlm1* [24]. On the other hand, there are cases where two *Avr* genes must act together to be able to trigger one *R* gene, the so-called two-gene-for-one-gene interaction. For example, *Rlm10*-mediated recognition can be triggered only if both *Avrlm10A* and *Avrlm10B* are present together [25]. Additionally, it was reported that the functional allele *Avrlm7* masks the recognition of *Avrlm9* and *Avrlm3* due to an epistatic interaction effect [26,27]. In *L. maculans*, fourteen *Avr* genes have been identified so far. Eight of them were cloned: *Avrlm1-L3* [28], *Avrlm2* [29], *Avrlm3* [26], *Avrlm4-7* [30], *Avrlm5-9* [27,31], *Avrlm6* [32], *Avrlm10* [25] and *Avrlm11* [33]. On the side of the host, in *B. napus*, an even larger number of corresponding major *R* genes were described, such as *Rlm1, Rlm2, Rlm3, Rlm4, Rlm5, Rlm6, Rlm7, Rlm8, Rlm9, Rlm10, Rlm11, RlmS (BLMR1.2), Rlm13, LepR1, LepR2, LepR3 (BLMR1.1)* and *LepR4*. Only three of them, *Rlm2, Rlm9* and *LepR3,* were cloned [24,34,35,36,37,38,39,40]. 

Sowing a specific commercial cultivar harboring a major *R* gene in a region over years results in high natural selection pressure. Hence, new *L. maculans* races evolve that can overcome the introduced major *R* gene. This is called a “boom and bust” cycle [5]. The amplitude of a “boom and bust” cycle of a major *R* gene differs among fungal phytopathogens. For *L. maculans*, several studies have documented the potential lifespan of a major *R* gene when intensively deployed under experimental conditions or at a commercial level. For example, Brun et al. reported that *Rlm6* turned ineffective after three growing seasons in field experiments [41]. On a commercial level, breakdowns of major *R* genes have been observed within three to five years after their introduction into the market in many oilseed rape-growing countries, such as the efficacy loss of *Rlm1* in France, *Rlm3* in western Canada and “sylvestris”-derived resistance, namely *Rlm1* and *LepR3*, in Australia [42,43,44].

Setting a strategic regional plan to rotate cultivars harboring major *R* genes is essential to expand the efficacy longevity of major *R* genes. The reason is that using qualitative resistance is only reasonable as long as the corresponding *Avr* gene is dominant in the population. However, a reliable plan for major *R* gene rotation requires regular updating of the *Avr* profile of regional *L. maculans* populations [45]. Monitoring of *Avr* gene frequencies is thus crucial for practical recommendations for farmers and breeders. 

The last study that investigated *Avr* gene frequencies in *L. maculans* populations in Germany was based on samples collected in the growing seasons of 2011 and 2012 [46]. Here, we aimed not only to update the *Avr* profile of *L. maculans* populations, but also to expand the range of the tested *Avr* genes by including *Avrlm6, Avrlm11, AvrlepR1, AvrlepR2* and *AvrlepR3,* which have not been investigated in Germany so far. In addition, special attention was given to the change in *Avrlm7* frequency, since *Rlm7* has been known as the most effective commercialized major *R* gene in Germany in recent years. Additionally, this study aimed to check whether *L. maculans* population mating types deviate from the hypothesized 1:1 ratio in northern and central Germany. 

## 2. Results

**Figure 1:**
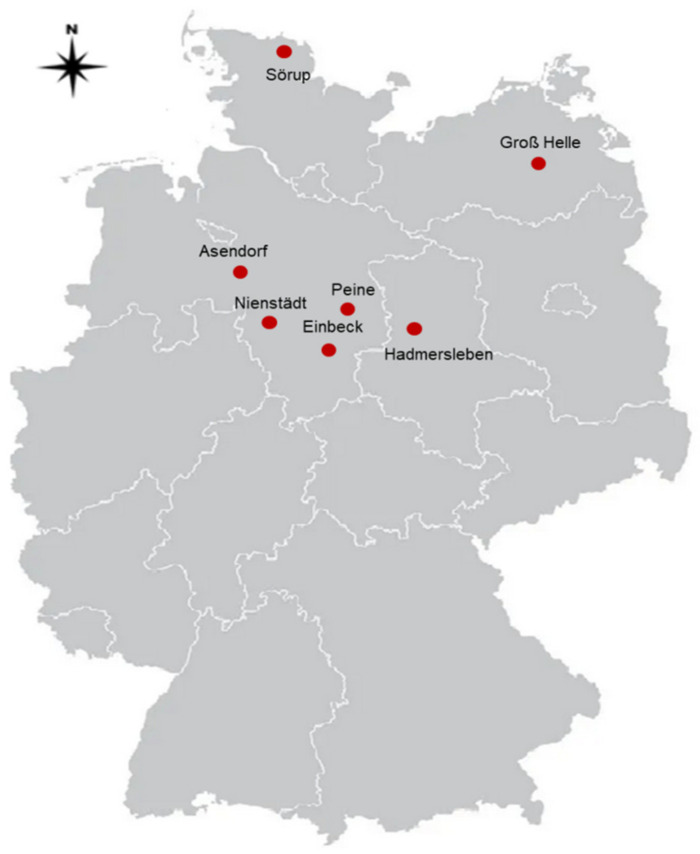
Field trial locations in central and northern Germany from which leaf samples with phoma lesions were collected to race type *L. maculans* populations (2017 to 2020).

Six hundred isolates were collected during three seasons from 2017 to 2020 from seven regions in four provinces in northern and central Germany (Figure 1). Using the primers ITS5/ITS4 targeting the internal transcribed spacer (ITS) regions, 26 isolates were identified as *L. biglobosa* while 574 isolates were assigned to *L. maculans.* In the pathogenicity test, the 26 *L. biglobosa* isolates caused only very small necrotic leaf spots with no pycnidia on the cotyledons. Additionally, in the susceptible check, cultivar Westar did not show more severe infection when inoculated with the *L. biglobosa* isolates. The 574 *L. maculans* isolates were further characterized to determine their races and their mating types.

### 2.1. Efficacy of Major Resistance Genes in German Fields

The 574 isolates assigned to *L. maculans* were used to monitor the *Avr* profile of the pathogen population and thus the *R* gene efficacies. Eleven avirulence genes were characterized (*Avrlm1, Avrlm2, Avrlm3, Avrlm4, Avrlm6, Avrlm7, Avrlm9, Avrlm11, AvrlepR1, AvrlepR2* and *AvrlepR3*), either phenotypically by performing cotyledon tests on an oilseed rape differential set or genotypically by using *Avr* gene-specific PCR primers.

**Figure 2:**
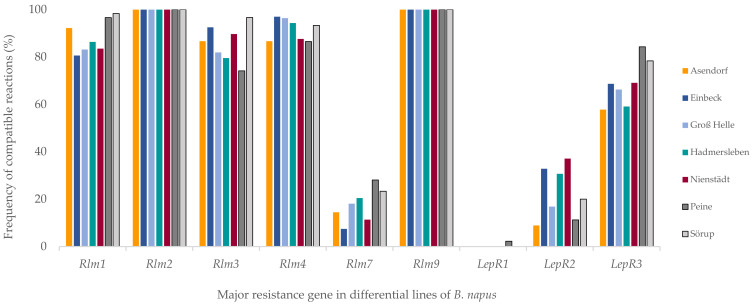
Virulence frequencies of *L. maculans* isolates originating from different fields tested on a *B. napus* differential set. Cotyledon tests were conducted with 574 isolates. Field sites and numbers of tested isolates: Hadmersleben, n = 88; Groß Helle, n = 83; Nienstädt, n = 97; Einbeck, n = 67; Sorüp = 60; Asendorf = 90, Peine, n = 89. Isolates were collected from 2017 to 2020.

Results of the pathogenicity test provide evidence that all the tested isolates were virulent on differential lines that harbored *Rlm2* and *Rlm9* (Figure 2). This shows that *Rlm2* is ineffective in the explored regions. However, the 100% virulence frequencies on the *Rlm9* differentials were expected since the trap variety NK Bravour harbors *Rlm9*. In contrast, Figure 2 showed that *AvrlepR1* proved to be the most abundant avirulence gene with 100% presence in all investigated regions, except in Peine, where two isolates virulent on the differential line Topas-*LepR1* were detected, representing 2% of Peine’s population and 0.3% of the whole isolate collection. 

There was a drastic increase in the frequency of *avrlm7* isolates (i.e., isolates virulent on *Rlm7*) demonstrated in this study when compared with the results of our previous work on *Rlm7* in Germany (2011–2012) [46]. A comparative analysis of both studies showed that the *avrlm7* isolate’s frequency increased within 5–7 years from 0.9% to 17.6% on average in all investigated regions. This study confirmed that *Rlm7* has become less effective in Germany since 2011–2012, only a few years after its commercial release. According to Figure 2, the percentage of *avrlm7* isolates ranged from 7% in Einbeck to 28% in Peine.

Frequencies of compatible interactions on *Rlm1, Rlm3, Rlm4* and *LepR3* varied from region to region. Phenotypical data revealed that the functional *Avrlm1* ranged from 2% to 19%, *Avrlm3* from 3% to 26%, *Avrlm4* from 3% to 13% and *LepR3* from 16% to 42%. These results indicate that the referred *R* genes are still partially effective. Interestingly, although the alternating functionality supposed due to the masking effect between *Avrlm7* and *Avrlm3* can be recognized in Figure 2, 17 *avrlm7* isolates out of 101 were virulent on the differential Topas-*Rlm3,* despite the absence of the functional *Avrlm7*. 

*LepR2* is also partially effective. However, with a frequency of functional *AvrlepR2* ranging from 63% to 91%, *LepR2* can be considered similarly effective as *Rlm7* in some regions, and thus, the two can be used in rotation with each other to avoid or delay the further resistance breakdown of both. The results in Figure 2 emphasize the importance of considering the regional differences for *L. maculans* management strategies. For example, Figure 2 shows that, while *LepR2* is more effective than *Rlm7* in Peine, the opposite is true in Einbeck and Nienstädt.

**Figure 3:**
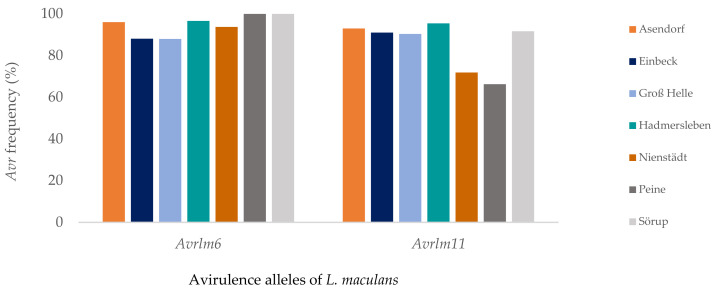
Frequencies of avirulence genes *Avrlm6* and *Avrlm11* in *L. maculans* isolates collected from different fields in Germany tested by PCR. In total, 574 isolates were tested. Field sites and numbers of tested isolates: Hadmersleben, n = 88; Groß Helle, n = 83; Nienstädt, n = 97; Einbeck, n = 67; Sorüp = 60; Asendorf = 90, Peine, n = 89.

Since we did not have access to *B. napus* lines harboring the major *R* genes *Rlm6* and *Rlm11*, we used specific PCR primers to test for *Avrlm6* and *Avrlm11*. To our knowledge, there are no reports from other research groups who have differential lines including *Rlm6* and *Rlm11* about masking effects of *Avr* genes that hinder the recognition of *Avrlm6* or *Avrlm11.* Additionally, it was reported that deletion is the major mechanism of gaining virulence in these *Avr* genes [33,47]. Therefore, we assumed that the results of the PCR avirulence gene tests would likely match the results of the phenotyping by cotyledon tests. Figure 3 shows that the frequency of *Avrlm6* ranged from 88% to 100%, while the frequency of *Avrlm11* was 72% to 95%. Consequently, it can be concluded that *Rlm6* and *Rlm11* are relatively effective, and their efficacies are comparable to that of *Rlm7*. However, it is noteworthy that there are no reports stating the introduction of these two genes into commercial cultivars in Germany. 

### 2.2. L. maculans Races in German Populations

In general, 52 races were described among the 574 *L. maculans* isolates collected from 7 sites in Germany. However, richness in races according to the Margalef index differed between regions. Table 1 illustrates that Nienstädt showed the highest race diversity with 25 races and a Margalef index of 5.25, whereas Sörup displayed the lowest race diversity with 14 races and a Margalef index of 3.18. 

The races presented in Table 2 are based on the phenotyping tests and the PCR tests of *Avrlm6* and *Avrlm11*. Since *LepR3* is assumed to interact with *Avrlm1*, it was not possible to ensure the presence of *AvrlepR3* distinctly from *Avrlm1* in isolates avirulent on both *LepR3* and *Rlm1*. Therefore, these isolates were marked with asterisks to draw attention to the possible redundance. Some researchers hypothesize that *Avrlm1* and *AvrlepR3* are identical, and that *AvrlepR3* is a hypothetical gene [48]. However, there were races in our tested collection that were virulent on *Rlm1* and avirulent on *LepR3*. This indicates that this assumption may not be true. 

The predominant race in all regions investigated was *Avrlm 6, 7, 11, AvrLepR 1, 2,* which represented 30.8% of the whole races in the tested German *L. maculans* population. The second most dominant race was *Avrlm6, 7, 11, AvrlepR1, 2, 3,* making up 11.8% of the population, and differing from the most dominant one by the presence of *AvrlepR3*. The third dominant race was *Avrlm6, 7, 11, AvrlepR1,* with a share of 11.5%, and the fourth ranking was *Avrlm3, 6, 11, AvrlepR1, 2,* at a rate of 7.1%. The four most dominant races together accounted for 61.3% of all races in the tested German population. An effective oilseed rape cultivar rotation should basically consider these most dominant races. 

**Figure 4:**
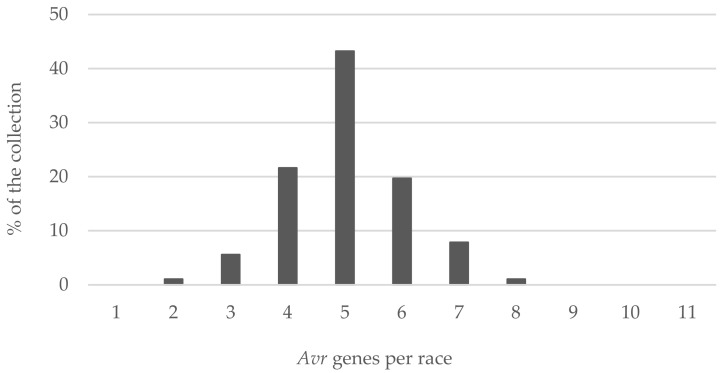
Frequencies of *Avr* gene complexity in races of the investigated German *L. maculans* population.

Isolates of the *L. maculans* population in the explored regions had between two and eight functional avirulence genes (Figure 4). Among them, 43% had five different *Avr* genes, 22% had four *Avr* genes and 20% had six *Avr* genes. Only a few isolates (1%) had two or eight *Avr* genes.

### 2.3. L. maculans Mating Type Ratio in German Fields

**Figure 5:**
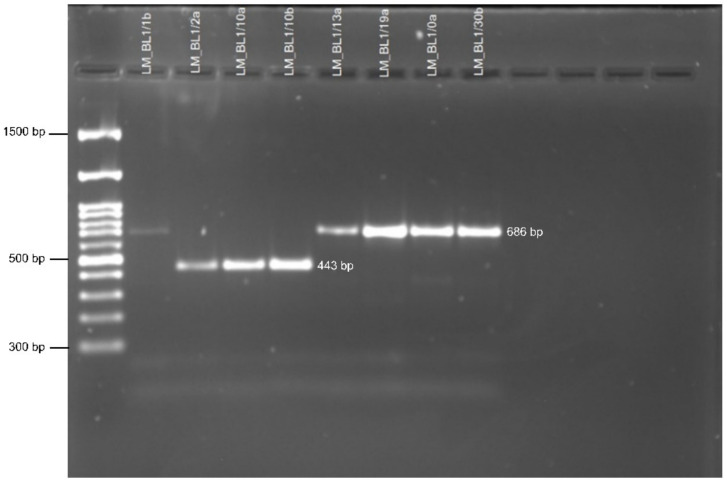
Agarose gel electrophoresis of the PCR-amplified mating type loci of *L. maculans* using a multiplex PCR system described by Cozijnsen and Howlett [11]. Three primers were used. The use of a common primer in combination with the *MAT1-1* locus-specific primer results in an amplicon of 686 bp, while the use of it in combination with the *MAT1-2* locus-specific primer affords an amplicon of 443 bp.

Mating types were defined using multiplex PCR in 562 isolates (Figure 5). Both idiomorphs of the pathogen existed in each region and Fisher’s exact test proved no significant departure from the 1:1 ratio of the two mating types (Table 3). This emphasizes the importance of the annual sexual reproduction in the lifecycle of *L. maculans* in the explored regions. In fact, primary infection in Germany depends on the spread of ascospores, a factor that enhances the ability of the fungus to adapt rapidly to new qualitative major *R* genes implemented in newly commercialized cultivars.

## 3. Discussion

This is the first study identifying the frequencies of *AvrlepR1*, *AvrlepR2*, *AvrlepR3*, *Avrlm6* and *Avrlm11* in the *L. maculans* population in German fields. Besides, it has updated the *Avr* profiles of *Avrlm1*, *Avrlm2*, *Avrlm3*, *Avrlm4* and *Avrlm7,* which have not been investigated in Germany since 2012 [46]. Cotyledon tests showed that 100% of the isolates were virulent on differential lines harboring *Rlm2*. The absence of *Avrlm2* was also described in northern Germany in isolates sampled in 2011–2012 [46]. Similar results have previously been found in France in 2000–2001 [49]. However, this situation is different on other continents. For example, in western Canada, a survey on samples collected in 2012–2014 showed that *Avrlm2* reached 80% [50]. All our sampled isolates were virulent on the *Rlm9* differential. This can be explained by the use of the *Brassica napus* trap cultivar NK-Bravour that carries the *Rlm9 R* gene. This led to a preselection of *avrlm9*-harboring isolates, whereas *Avrlm9*-harboring isolates were counter-selected. 

Epistatic effects, as a mechanism for evading recognition, were reported by *Avrlm7* toward *Avrlm3* [26] and *Avrlm9* [27]. Indeed, the results of our study supported that the presence of the functional *Avrlm7* masked the recognition of *Avrlm3*. Investigations of epistatic mechanisms of *Avrlm7* toward *Avrlm3* and *Avrlm9* revealed that this suppression was caused by neither stopping the expression of *Avrlm3* and *Avrlm9*, nor a physical interaction of the *Avr* effector proteins of *Avrlm3*, *Avrlm9* and *Avrlm4*–*7* [27,51]. Using protein structure approaches, it was demonstrated that the three effector proteins of these genes belong to a new family of effectors, called *Leptosphaeria* AviRulence-Supressing effectors (LARS). LARS are structurally analogue effectors that differ in their amino acid identities, although they share common targets in the plants [52]. 

In our study, several compatible interactions on the *Rlm3* differential lines were detected among isolates lacking the functional *Avrlm7.* This indicates that although *Avrlm7* masks the recognition of *Avrlm3*, its absence does not mean that the pathogen has no other means to avoid recognition. A variety of virulence-gain mechanisms in plant pathogens have been described, such as deletion of the *Avr* gene, point mutations that allow the pathogen to avoid recognition despite the presence of the *Avr* gene, amino acid substitution and masking the *Avr* protein through another *Avr* protein [26,47,53]. Gene silencing of *Avrlm3* proved that this *Avr* gene is crucial in *L. maculans* pathogenicity and has an important effect on its lifecycle in *B. napus* [51]. Thus, deletion of the gene can be ruled out by elucidating the virulence of *avrlm7* isolates on *Rlm3*. Plissonneau et al. explained the virulence of *avrlm7* isolates toward *Rlm3* by the high allelic polymorphism of *Avrlm3*, which allows a high level of possible protein isoforms. Therefore, it can be speculated that the alternative mechanism used by the pathogen when the epistatic effect disappears is to substitute an amino acid in the effector protein to allow a so-called “camouflage” type of escaping recognition [51]. Setting strategies for *R* gene management based on restoring the efficacy of *Rlm9* and *Rlm3* by losing the functional *Avrlm7* could mislead farmers into reducing caution in *Rlm7* deployment.

Our results showed that major *R* genes *Rlm1, Rlm3, Rlm4* and *LepR3* are not able to provide sufficient resistance against phoma stem canker in German fields. In the explored regions, *Avrlm4* isolate frequencies ranged from 3% to 13%. This is quite close to the rate described in a large-scale survey throughout France in 2000–2001 (0–19.5%) [49]. *Avrlm4*-harboring isolates turn virulent when glycine at position 120 in the *Avr* protein is substituted with arginin [54], while *Avrlm1* and *AvrlepR3*, which are suspected to be alleles of the gene *Avrlm1-L3*, turn virulent mainly due to deletion of the whole gene [55,56]. Our results showed that *Avrlm1* frequency ranged from 2% to 19%. A rapid adaptation of the *L. maculans* population toward *Rlm1* was noticed in France, where the rate of *Avrlm1*-harboring isolates made up 83% of the population in 1997–1998 and decreased dramatically to less than 13% in 1999–2000 [42]. Similarly, in Canada, *Avrlm1* frequency did not exceed 5% [45]. In Australia, the efficacy of *Rlm1* resistance in cultivar Surpass 400 notably decreased within three years after commercial release [23], as well as the efficacy of *LepR3*. This is explained by the fact that *Avrlm1* has dual specificity and can trigger both *R* genes: *Rlm1* and *LepR3* [24]. It can be concluded that wherever *Rlm1* efficacy is broken, *LepR3’s* lifespan is shorter than that of other *R* genes, in which the corresponding *Avr* gene does not have dual specificity.

We identified a dramatic increase in the frequency of *avrlm7* isolates compared with the 2011–2012 season. The frequency of *avrlm7* isolates increased within five to seven years from 0.9% to 17.6% in fields located in central and northern Germany, and the highest presence of them was in Peine, where the frequency reached 28%. Winter and Koopmann stated that *Rlm7* was the only still effective major *R* gene used in commercial cultivars in Germany [46]. The potential speed of *Rlm7* breakdown was experimentally tested by applying maximum pressure on the *L. maculans* population through sowing cultivars with *Rlm7* for four years, without applying crop rotation or ploughing in the crop debris. Under these experimental conditions, the results revealed that *avrlm7* isolates frequency increased from 0 to 36% within 4 years [54]. In 2006, a study at the European level showed that the *Avrlm7* frequency was 100% in France, Germany, Sweden and Poland [57]. However, the intense deployment of *Rlm7* resulted in a rapid reduction of *Avrlm7* not only in Germany, but also in France. There, *Rlm7* was introduced commercially in 2004. By 2013, the frequency of *Avrlm7* isolates reached an average of 19.5% and a maximum of 45%, depending on the region [58]. In contrast, *Rlm7* is still effective in Canada, with an *Avrlm7* frequency exceeding 98% in 2018–2019 [45]. Hence, the present study calls for a more cautious deployment of *Rlm7,* especially in Europe. 

Some major *R* genes in oilseed rape were derived from related *Brassica* species. For instance, while *Rlm11*, *LepR1, LepR2* and *LepR3* were introduced into *B. napus* from *B. rapa* [33,34,35], *Rlm6* originated from *B. juncea* [59]. Our survey provided strong evidence that *LepR1* is the most effective major *R* gene in Germany, with 100% frequency of the isolates harboring *AvrlepR1* in all regions investigated, except in Peine, where 2% of the isolates were virulent on *LepR1*. Although the proportion of the virulent isolates toward *LepR1* was very low, and such isolates were found only in one region, the former observation of the rapid decline in efficacy of *Rlm7* within 5–7 years should prompt to take measures to preserve the efficacy of *LepR1*. 

In France, *AvrlepR1, AvrlepR2, Avrlm6* and *Avrlm11* frequencies in the *L. maculans* population were shown to still be high enough to control the disease in the field [60]. This is in line with our findings in Germany. A significant constraint in the targeted use of *R* genes in the field is a lack of knowledge about their presence in several commercial cultivars registered in Germany. *LepR2*, which is believed to be the same gene or an allelic form of *RlmS*, was reported in cultivars recently registered in France and Germany [61]. Considering the frequency of *avrlepR2* in Germany of 9–37%, as shown in our study, an annual monitoring seems necessary in regions where cultivars harboring *LepR2* are grown. This may help to avoid a situation such as that in Canada, where *AvrlepR1*, *AvrlepR2* and *AvrlepR3* occur already at low frequencies of ca. 19%, 5% and 5–28%, respectively [45].

The major mechanism of virulence gain of *Avrlm6* is a deletion of the gene [47]. Van de Wouw et al. studied isolates collected in Australia over two decades (from 1987 to 2017) and observed fluctuating frequencies of *Avrlm6* from 21% to 80% depending on the year. This behavior appeared to be independent from the commercial introduction of *Rlm6* in Australia in 2010 [62]. Hence, the study speculated that *Avrlm6* frequency might also be influenced by the intensive deployment of other major *R* genes in Australia [62]. In France, *Rlm6* was not introduced commercially after the French National Institute for Agricultural Research (INRA) decided to keep it as a research tool [41]. As a result, *Avrlm6* frequency was 100% in France [49], similar to Canada, where *Avrlm6* frequency exceeds 98% [45]. In our study, a fluctuation in frequency between 88% and 100% depending on the region was observed, however the use of *Rlm6* in Germany is not clear. 

*Avrlm11* has been reported to occur in high frequencies in many regions of the world. For instance, its frequency was >95% in France and 100% in Canada [33,45]. In our study, the rate of isolates harboring *Avrlm11* ranged from 66% to 95%. Despite the high frequency of *Avrlm11*, it was found to be located on a dispensable mini-chromosome of *L. maculans* that was occasionally lost by meiosis. Thus, the loss of *Avrlm11* is related to the loss of this mini-chromosome in *L. maculans.* [33]. In spite of its dispensability, this chromosome turned out to be influential for the viability of ascospores, and thus, its loss has fitness costs [33]. 

The race structure in our study was described based on the phenotypic analysis of eight *Avr* genes and the PCR assays for *Avrlm6* and *Avrlm11*. Assuming each of the 10 tested *Avr* genes has a minimum of 2 alleles, the theoretical number of combinations would be 1024 (i.e., 2^10^). This shows the theoretical potential of the pathogen to break resistance. In our study, we were able to determine 52 races, the majority of which had a complexity of five *Avr* genes. Race complexity depicts the range of the available effective major *R* genes for setting management strategies for resistant cultivar rotation in a region [45]. In general, we found, according to our isolate collection, that the major *R* genes that provide sufficient resistance in German oilseed rape fields are *LepR1, LepR2, Rlm6, Rlm11* and *Rlm7,* while 61.3% of the isolates can equally infect cultivars harboring *Rlm1, Rlm2* and *Rlm4.* This is consistent with our previous investigation in Germany [46]. Combining quantitative resistance with race-specific resistance can be important to expand the durability of major *R* genes. It was demonstrated that *Rlm6* in a cultivar with a quantitative resistance background preserved its efficacy two years longer than in a cultivar with a susceptible background [41]. 

Pathogens with a high evolutionary potential are expected to overcome genetic resistance more rapidly [5]. It is believed that there are two important factors that enhance the evolutionary potential of *L. maculans*: first, the mixed reproduction systems, and second, the dispersal mechanisms. The sexual reproduction is a milestone for producing variations in the population, while the wind dispersal of spores spread the evolving races effectively over several kilometers [54,63]. However, in seasons when the environmental conditions do not favor sexual reproduction, asexual reproduction would be dominant, and thus, the ratio of idiomorphs might change. For example, in some Canadian regions, samples collected in 2010 and 2015 showed that the mating type *MAT1-2* was significantly more dominant than *MAT1-1* [64]. In our study, we showed that the idiomorphs of *L. maculans* in Germany did not significantly deviate from the ratio 1:1. This is consistent with the ratio observed in France in isolates collected in 2000–2003 [65] and in Canada in samples from 2011–2014 [64], which indicates similar lifecycles of this pathogen in different regions in the world and a central role of the sexual outcrossing in forming the population of the pathogen.

Overall, we comprehensively compared the effectiveness of major *R* genes in different countries. We found similarities in the *R* gene efficacies throughout Germany’s neighboring countries, but increasingly significant differences the further those countries were from our study region. This may be related to the fact that the pathogen mainly spreads through wind-borne ascospores that can fly up to several kilometers and stay viable for six weeks [1]. We therefore recommend the consideration of geographical factors for the successful management of blackleg disease.

## 4. Materials and Methods

### 4.1. Sampling and Isolation of Leptosphaeria maculans

To explore the race spectrum of *Leptosphaeria maculans* in Germany, samples were collected from plots sown with the winter oilseed rape cultivar NK-Bravour (Syngenta Seeds GmbH, Bad Salzuflen, Germany). Plants grown in these plots were used as *L. maculans* trap plants, since this cultivar only harbors the major *R* gene *Rlm9* (H. Uphoff and M. Gundemann, Syngenta Seeds, pers. comm) [66]. The field trials were established in cooperation with breeding companies in seven regions of four provinces in Germany. Four fields were located in Lower Saxony in Einbeck (KWS), Nienstädt (Bayer CropScience), Peine (Limagrain) and Asendorf (DSV), one field in Saxony Anhalt in Hadmersleben (Syngenta), one field in Mecklenburg Western Pomerania in Groß Helle (NPZ) and one field in Schleswig Holstein in Sörup (BASF). Samples were collected in seasons 2017/2018, 2018/2019 and 2019/2020 (Table 4). 

Leaf samples with characteristic phoma lesions were collected in autumn at growth stage BBCH 18 and in spring at growth stage BBCH 30 [67]. Samples were dried and stored at 4 °C until they were used for isolation. Isolation was conducted by incubating a dried leaf segment with a lesion in a humid chamber at room temperature to induce conidiation. Single pycnidium isolates were prepared by plating spores on synthetic Nutrient-Poor Agar (SNA) medium amended with 200 ppm streptomycin under sterile conditions. Petri dishes were incubated under NUV light at 20 °C for six days. Afterwards, a mycelial plug was transferred to a V8-juice medium supplemented with 200 ppm streptomycin and incubated for 10–14 days under the same conditions. Subsequently, spore suspensions were prepared and adjusted to a density of 1 × 10^7^ spores/mL using a hemocytometer. Spore suspensions were stored at −20 °C and thawed just before they were used for inoculation.

To characterize *L. maculans* isolates, cotyledon tests were applied using a differential set of *B. napus* genotypes harboring the major *R* genes *Rlm1*, *Rlm2*, *Rlm3*, *Rlm4*, *Rlm7*, *Rlm9*, *LepR1*, *LepR2* and *LepR3*. The cultivars Westar and Topas DH16516 had no major *R* genes and were used as susceptible controls (Table 5). 

Seeds were sown in trays filled with potting soil and covered with transparent plastic plates to keep a high level of humidity. After three days, when seeds germinated, the covers were taken off. On the sixth day, seedlings were transplanted in multi-pot propagation trays with a 3:3:1 mixture of garden soil, compost and sand. On day seven, seedlings were inoculated with 10 µL of spore suspension placed on each lobe of the cotyledons after injuring it with a needle. Seedlings were then put under controlled conditions of 16:8 h light (day/night) at 20 °C. For each isolate and differential line, eight seedlings were inoculated. Symptoms were evaluated 14 days post-inoculation according to the IMASCORE rating scale, where class one shows typical hypersensitive reactions and class six reflects tissue collapse with sporulation. Classes one to three are considered as incompatible reactions while classes four to six are noted as compatible ones [72].

### 4.2. DNA Extraction and PCR Assays

DNA templates were prepared using a simple boiling DNA extraction method. Shortly, 100 µL of spore suspension (10^7^ spores/mL) was centrifuged at 16,000× *g* for 10 min. Afterwards, the supernatant was discarded carefully to keep the pellet. Fifty microliters of Tris (10 mM) was added to the pellet and homogenized by vortexing. The suspension was exposed to an ultrasound for 5 s and then transferred to a water bath at 98 °C for 10 min. Subsequently, the tubes were put on ice for 10 min. Before applying the PCR tests, the quality and quantity of the extracts were checked using agarose gel electrophoresis (0.8% (*w*/*v*)) supplied with Midori Green (NIPPON Genetics Europe GmbH). Five µL of DNA was mixed with two µL of the loading dye (100 mM EDTA, 50% (*v*/*v*) glycerol and 0.025% (*w*/*v*) bromophenol-blue) and run in TBE buffer at 3 V/cm for 60 min. DNA quantity was afterwards compared with a standard Lambda phage DNA of concentrations ranging from 150 to 35.5 ng. The concept of releasing DNA by boiling the cells was also used by Adwan [73]. 

To distinguish *L. maculans* and *L. biglobosa,* we amplified the internal transcribed spacer (ITS) regions of the pathogen using ITS4 and ITS5 primers (Table 6). The steps of the 35 PCR thermal cycles are described in Table 7. *Avrlm6* and *Avrlm11* were amplified using the primer pairs listed in Table 6 in 40 and 30 thermal cycles, respectively. PCR profiles are shown in Table 7. In general, the end volume of a PCR reaction was 25 µL, of which 7 µL was DNA extract. The mixture contained 1 µM of each primer pair, except for the mating type primers, where 0.67 µM of each of the three primers were used. The concentration of the nucleoside triphosphates (dNTPs) was 0.2 mM, and 1 unit of the FastGene Taq DNA polymerase (NIPPON Genetics Europe GmbH) was added. The reaction was conducted in a buffer containing 1.5 mM of MgCl_2_. PCR reactions were conducted in a T Professional Basic Gradient thermal cycler (Biometra, Göttingen, Germany).

To visualize the PCR products, 5 µL was mixed with 2 µL of the loading dye described above. The mixture was loaded on 1% agarose gel supplied with Midori Green. Electrophoresis was run at 3 V/cm for 60 min for all PCR products, except for the mating type tests, where the electrophoresis was run for at least two hours. Mating types of *L. maculans* were defined using a multiplex PCR, as described by Cozijnsen and Howlett [11]. An isolate of *L. maculans* has a single mating locus (*MAT* locus) and one of the two loci *MAT1-1* or *MAT1-2*. The common primer for the *MAT* locus in combination with the *MAT1-1*-specific primer resulted in an amplicon of 686 bp, while the common primer in combination with the *MAT1-2*-specific primer afforded an amplicon of 443 bp. 

### 4.3. Statistical Analysis

For phenotypic characterization of the *L. maculans* population, the numbers of compatible (susceptible) reactions observed in the cotyledon tests were represented relative to the number of isolates collected per region. Similarly, *Avrlm6* and *Avrlm11* that produced bands in PCR tests were represented as the proportion of the total number of isolates analyzed per region. The Margalef index (D_Mg_) was calculated using the following formula:

D_Mg_ = (S − 1)/Ln(N)

where S is the number of races per region, and N is the number of *L. maculans* isolates in the corresponding region.

Fisher’s exact test χ^2^ was applied using the software R [76] to test the null hypothesis that the mating types of *L. maculans* in German fields do not deviate from the ratio 1:1 at a confidence level of 95%.

## 5. Conclusions

In this study, the *L. maculans* population in German oilseed rape fields was characterized by their races. The resulting *Avr* profile of the pathogen provides an important basis for evaluating the state of efficacy of major *R* genes used in commercial cultivars. Such knowledge is a prerequisite for establishing a management strategy with the aim of protecting *R* genes from a premature decline in efficacy and for expanding their durability in practical use. The rapid breakdown of major *R* genes identified in this study strongly implies the need for combining resistant cultivars with other control measures. Considering the long breeding cycles of 7–10 years required to have a new major *R* gene established in a registered new cultivar, it is important to combine quantitative resistance with major *R* genes in order to achieve more durable and sustainable disease control. The similarities observed in *Avr* profiles of *L. maculans* between France and Germany suggest that such management strategies can be valuable on a broader geographical scale. In addition, different *Avr* profiles existing in European and Canadian oilseed rape fields imply that transfer of races between continents by seed trade must be avoided. Hence, we emphasized the importance of considering *L. maculans* in the phytosanitary measures, ensuring international clean seed pathways. 

## Figures and Tables

**Table 1:** Number of *L. maculans* and *L. biglobosa* isolates and Margalef index indicating the local population diversity of *L. maculans* races at the different sites.

Site	Province	No. of Isolates	*L. biglobosa*	No. of Races	Margalef Index
Nienstädt	Lower Saxony	99	2	25	5.25
Groß Helle	Mecklenburg-Western Pomerania	83	0	21	4.53
Peine	Lower Saxony	97	8	20	4.23
Einbeck	Lower Saxony	71	4	16	3.57
Asendorf	Lower Saxony	100	10	18	3.56
Hadmersleben	Saxony Anhalt	90	2	16	3.35
Sörup	Schleswig-Holstein	60	0	14	3.18

**Table 2:** Race spectrum of *L. maculans* populations from seven field sites in Germany collected from 2017 to 2020. Races are described based on phenotypic characterization of *Avrlm1, Avrlm2, Avrlm3, Avrlm4, Avrlm7*, *AvrlepR1, AvrlepR2* and *AvrlepR3. Avrlm6* and *Avrlm11* were characterized based on specific PCR primers.

*L. maculans* Races	Percentage of Total Number of Isolates Collected Per Region							
	Asendorf	Einbeck	Groß Helle	Hadmersleben	Peine	Sörup	Nienstädt	Total
*Avrlm6, -7, -11, AvrlepR1, -R2*	31	25	40	28	29	43	16	30.8
*Avrlm6, -7, -11, AvrlepR1, -R2, -R3*	21	7	10	17	4	10	9	11.8
*Avrlm6, -7, -11, AvrlepR1*	5	15	7	19	4	17	13	11.5
*Avrlm3, -6, -11, AvrlepR1, -R2*	5	7	10	4	12	2	6	7.1
*Avrlm1, -6, -7, -11, AvrlepR1, -R2, -R3* *	4	11	4	1	0	2	4	3.7
*Avrlm6, -7, AvrlepR1, -R2*	0	0	2	0	9	0	7	3.1
*Avrlm4, -6, -7, -11, AvrlepR1, -R2, -R3*	4	0	1	3	3	2	3	2.6
*Avrlm4, -6, -7, -11, AvrlepR1, -R2*	6	0	0	0	3	0	3	2.1
*Avrlm6, -7, AvrlepR1*	1	1	0	0	2	2	7	2.1
*Avrlm3, -6, -11, AvrlepR1, -R2, -R3*	3	0	2	3	1	0	1	1.7
*Avrlm6, -11, AvrlepR1, -R2*	2	0	0	0	1	10	0	1.6
*Avrlm7, AvrlepR1, -R2*	0	6	0	0	0	0	5	1.6
*Avrlm1, -6, -7, -11, AvrlepR1, -R3* *	0	0	1	3	0	0	4	1.4
*Avrlm1, -6, -7, AvrlepR1, -R2, -R3* *	0	0	5	1	2	0	1	1.4
*Avrlm3, -6, AvrlepR1, -R2*	0	0	0	0	7	0	1	1.4
*Avrlm3, -6, -11, AvrlepR1*	0	0	1	6	1	0	1	1.4
*Avrlm7, -11, AvrlepR1, -R2*	1	0	5	1	0	0	1	1.2
*Avrlm1, -3, -6, -11, AvrlepR1, -R2, -R3* *	0	0	1	6	0	0	0	1.0
*Avrlm1, -4, -6, -7, -11, AvrlepR1, -R2, -R3* *	1	1	1	0	1	0	2	1.0
*Avrlm4, -6, -7, AvrlepR1, -R2*	0	0	0	0	4	0	2	1.0
*Avrlm6, -7, -11, AvrlepR1, -R3*	2	4	0	1	0	0	0	1.0
*Avrlm3, -6, AvrlepR1, -R2, -R3*	1	0	0	0	1	3	0	0.7
*Avrlm7, AvrlepR1*	0	3	0	0	0	0	2	0.7
*Avrlm7, AvrlepR1, -R2, -R3*	0	3	0	2	0	0	0	0.7
*Avrlm4, -6, -11, AvrlepR1, -R2*	0	0	0	0	1	3	0	0.5
*Avrlm7, AvrlepR1, -R3*	0	0	1	0	0	0	2	0.5
*Avrlm7, -11, AvrlepR1*	0	3	1	0	0	0	0	0.5
*Avrlm1, -3, -6, -11, AvrlepR1, -R3* *	0	0	2	0	0	0	0	0.3
*Avrlm1, -4, -6, -7, -11, AvrlepR1, -R3* *	0	0	0	0	0	0	2	0.3
*Avrlm1, -6, -7, -11, AvrlepR1*	0	3	0	0	0	0	0	0.3
*Avrlm1, -6, -7, AvrlepR1, -R3* *	0	0	0	0	0	0	2	0.3
*Avrlm3, -6, AvrlepR1*	0	0	0	0	1	0	1	0.3
*Avrlm6, -7*	0	0	0	0	2	0	0	0.3
*Avrlm6, -7, AvrlepR1, -R2, -R3*	0	0	0	0	2	0	0	0.3
*Avrlm1, -3, -6, AvrlepR1, -R2, -R3* *	0	0	0	1	0	0	0	0.2
*Avrlm1, -4, -7, -11, AvrlepR1, -R2, -R3* *	0	0	1	0	0	0	0	0.2
*Avrlm1, -4, -7, AvrlepR1, -R2, -R3* *	1	0	0	0	0	0	0	0.2
*Avrlm1, -7, -11, AvrlepR1, -R2, -R3* *	0	1	0	0	0	0	0	0.2
*Avrlm1, -7, -11, AvrlepR1, -R3* *	0	0	1	0	0	0	0	0.2
*Avrlm1, -7, AvrlepR1, -R2, -R3* *	0	1	0	0	0	0	0	0.2
*Avrlm1, -7, AvrlepR1, -R3* *	0	0	0	0	0	0	1	0.2
*Avrlm3, AvrlepR1, -R2*	1	0	0	0	0	0	0	0.2
*Avrlm3, -11, AvrlepR1*	0	0	1	0	0	0	0	0.2
*Avrlm4, -6, -7, -11, AvrlepR1*	0	1	0	0	0	0	0	0.2
*Avrlm4, -6, -11, AvrlepR1, -R2, -R3*	0	0	0	0	0	2	0	0.2
*Avrlm4, -6, -7, Avrlm11, AvrlepR1, -R3*	0	0	0	1	0	0	0	0.2
*Avrlm6, -7, AvrlepR1, -R3*	0	0	0	0	0	2	0	0.2
*Avrlm6, -11, AvrlepR1, -R2, -R3*	0	0	0	0	0	2	0	0.2
*Avrlm6, -11, AvrlepR1*	0	0	0	0	0	0	1	0.2
*Avrlm7, -11, AvrlepR1, -R2, -R3*	0	0	1	0	0	0	0	0.2
*Avrlm6, AvrlepR1, -R2, -R3*	0	0	0	0	0	2	0	0.2
*Avrlm3, AvrlepR1, -R2, -R3*	1	0	0	0	0	0	0	0.2

* Since the major *R* gene in Topas-*LepR3* can be triggered by both *Avrlm1* and *AvrlepR3*, it was not possible to ensure the presence of *AvrlepR3* distinctly from *Avrlm1* in isolates avirulent on both *LepR3* and *Rlm1*. Therefore, such races are marked with an asterisk.

**Table 3:** Proportion of mating types of *L. maculans* in an isolate collection from different regions in central and northern Germany. Multiplex PCR was used to define the idiomorphs of the pathogen. Fisher’s exact test showed no significant deviation from the 1:1 ratio of the mating types (*p* = 0.05).

Site	No. of Isolates	*MAT1-1* (%)	*MAT1-2* (%)
Nienstädt	96	47	53
Groß Helle	73	44	56
Peine	89	42	58
Einbeck	65	45	55
Asendorf	94	56	43
Hadmersleben	88	48	52
Sörup	*57*	37	63

**Table 4:** Sampling seasons, regions and the numbers of *L. maculans* isolates.

Season	Region	Province	No. of Isolates
2017–2018	Einbeck	Lower Saxony	71
2017–2018	Nienstädt	Lower Saxony	99
2017–2018	Hadmersleben	Saxony Anhalt	90
2017–2018	Groß Helle	Mecklenburg-Western Pomerania	83
2018–2019	Sörup	Schleswig-Holstein	60
2019–2020	Peine	Lower Saxony	97
2019–2020	Asendorf	Lower Saxony	100
		Sum	600

**Table 5:** Differential sets of *B. napus* cultivars or introgression lines used for race typing of *L. maculans* isolates.

Cultivar/Line	*R* gene	References
Westar ^a^	No *R* gene	Balesdent et al., 2002 [68]
Columbus ^a^	*Rlm1, Rlm3*	Balesdent et al., 2006 [49]
Bristol ^a^	*Rlm2, Rlm9*	Balesdent et al., 2006 [49]
02–22-2-1 ^a^	*Rlm3*	Delourme, 2012 [69]
Jet Neuf ^a^	*Rlm4*	Balesdent et al., 2006 [49]
01-23-2-1 ^a^	*Rlm7*	Delourme, 2012 [69]
Caiman ^a^	*Rlm7*	Marcoft et al., 2012 [70]
Goéland ^a^	*Rlm9*	Balesdent et al., 2006 [49]
Topas DH16516 ^b^	No *R* gene	Larkan et al., 2016 [71]
Topas-*Rlm1* ^b^	*Rlm1*	Larkan et al., 2016 [71]
Topas-*Rlm2* ^b^	*Rlm2*	Larkan et al., 2016 [71]
Topas-*Rlm3* ^b^	*Rlm3*	Larkan et al., 2016 [71]
Topas-*Rlm4* ^b^	*Rlm4*	Larkan et al., 2016 [71]
Topas-*LepR1* ^b^	*LepR1*	Larkan et al., 2016 [71]
Topas-*LepR2* ^b^	*LepR2*	Larkan et al., 2016 [71]
Topas-*LepR3* ^b^	*LepR3*	Larkan et al., 2016 [71]

^a^ Provided by Dr. R. Delourme, Institute for Genetics, Environment and Plant Protection, INRA, Rennes, France. ^b^ Provided by Dr. Hossein Borhan and Dr. Nicholas Larkan, Agriculture and Agri-Food Canada, Saskatoon, Canada.

**Table 6:** Sequences of primers used in this study.

Primer	Sequence (5 to 3)	References
*Avrlm6*-F	TCAATTTGTCTGTTCAAGTTATGGA	Fudal et al., 2009 [74]
*Avrlm6*-R	CCAGTTTTGAACCGTAGAGGTAGCA	Fudal et al., 2009 [74]
*Avrlm11*-F	TGCGTTTCTTGCTTCCTATATTT	Balesdent et al., 2013 [33]
*Avrlm11*-R	CAAGTTGGATCTTTCTCATTCG	Balesdent et al., 2013 [33]
*MAT* Locus	TGGCGAATTAAGGGATTGCTG	Cozijnsen and Howlett, 2003 [11]
*MAT1-1*	CTCGATGCAATGTACTTGG	Cozijnsen and Howlett, 2003 [11]
*MAT1-2*	AGCCGGAGGTGAAGTTGAAGCCG	Cozijnsen and Howlett, 2003 [11]
ITS4	TCCTCCGCTTATTGATATGC	White et al., 1990 [75]
ITS5	GGAAGTAAAAGTCGTAACAAGG	White et al., 1990 [75]

**Table 7:** Profiles of the individual PCR assays.

Target	Initial Denaturation	Denaturation	Annealing	Extension	No. of Cycles
ITS	95 °C	94 °C	57 °C	72 °C	35
*Avrlm6*	95 °C	94 °C	60 °C	72 °C	40
*Avrlm11*	95 °C	94 °C	59 °C	72 °C	30
*MAT1-1*/*MAT1-2*	95 °C	94 °C	60 °C	72 °C	35

## Data Availability

Not applicable.
